# Advancing Biomarker Discovery and Therapeutic Targets in Duchenne Muscular Dystrophy: A Comprehensive Review

**DOI:** 10.3390/ijms25010631

**Published:** 2024-01-03

**Authors:** Monica Molinaro, Yvan Torrente, Chiara Villa, Andrea Farini

**Affiliations:** 1 Neurology Unit, Fondazione IRCCS Cà Granda Ospedale Maggiore Policlinico, Via Francesco Sforza 35, 20122 Milan, Italy; monica.molinaro@studenti.unimi.it (M.M.); yvan.torrente@unimi.it (Y.T.); 2 Stem Cell Laboratory, Dino Ferrari Center, Department of Pathophysiology and Transplantation, University of Milan, 20100 Milan, Italy; chiara.villa2@unimi.it

**Keywords:** Duchenne muscular dystrophy, Tdark gene, gut microbiota, immunity, biomarker

## Abstract

Mounting evidence underscores the intricate interplay between the immune system and skeletal muscles in Duchenne muscular dystrophy (DMD), as well as during regular muscle regeneration. While immune cell infiltration into skeletal muscles stands out as a prominent feature in the disease pathophysiology, a myriad of secondary defects involving metabolic and inflammatory pathways persist, with the key players yet to be fully elucidated. Steroids, currently the sole effective therapy for delaying onset and symptom control, come with adverse side effects, limiting their widespread use. Preliminary evidence spotlighting the distinctive features of T cell profiling in DMD prompts the immuno-characterization of circulating cells. A molecular analysis of their transcriptome and secretome holds the promise of identifying a subpopulation of cells suitable as disease biomarkers. Furthermore, it provides a gateway to unraveling new pathological pathways and pinpointing potential therapeutic targets. Simultaneously, the last decade has witnessed the emergence of novel approaches. The development and equilibrium of both innate and adaptive immune systems are intricately linked to the gut microbiota. Modulating microbiota-derived metabolites could potentially exacerbate muscle damage through immune system activation. Concurrently, genome sequencing has conferred clinical utility for rare disease diagnosis since innovative methodologies have been deployed to interpret the functional consequences of genomic variations. Despite numerous genes falling short as clinical targets for MD, the exploration of Tdark genes holds promise for unearthing novel and uncharted therapeutic insights. In the quest to expedite the translation of fundamental knowledge into clinical applications, the identification of novel biomarkers and disease targets is paramount. This initiative not only advances our understanding but also paves the way for the design of innovative therapeutic strategies, contributing to enhanced care for individuals grappling with these incapacitating diseases.

## 1. Introduction

Muscular dystrophies (MDs) represent a group of disorders characterized by primary skeletal muscle wasting and the subsequent emergence of co-morbidities such as inflammation, mitochondrial dysfunction, and metabolic irregularities. These conditions predominantly result from mutations in proteins that connect the cytoskeleton to the basal lamina [1].

Duchenne muscular dystrophy (DMD), the most prevalent form of muscular dystrophy, is a genetic disorder stemming from mutations in the dystrophin gene. Dystrophin deficiency leads to plasma-membrane instability, causing myofiber necrosis and muscle weakness [2]. The absence of dystrophin disrupts the contraction machinery, and the continuous degeneration/regeneration cycles in dystrophic muscles lead to persistent muscular injury and inhibition of regenerative potential caused by the depletion of satellite cells. This scenario culminates in the disruption of interactions between ion channels and components of the dystrophin glycoprotein complex, resulting in the dysfunction of transient receptor potential canonical (TRPC) and cardiac L-type calcium channels [3] as well as potassium and sodium channels, which contribute to developing cardiomyopathy [4].

All these events are exacerbated by alterations in metabolism and high energy needs that dramatically worsen myonecrosis, leading to fibrosis and inflammation. Inflammatory markers and specific lymphocyte subsets have been identified in the blood/muscles of DMD patients. Additionally, T lymphocytes from the murine model of DMD (mdx mouse) induce muscular damage when injected into healthy murine muscle [5].

Skeletal muscle inflammation in mdx mice follows a well-defined timeline, commencing early in the first two weeks of life, peaking at 6–8 weeks, and diminishing by 12 weeks. Following muscle damage and altered calcium influx, the initiation of the inflammatory phenotype is triggered by damage-associated molecular patterns (DAMPS) that activate neutrophils by means of specific membrane markers such as Toll-like receptors (TLRs) and macrophage-1 antigen (Mac-1). These cells recruit macrophages, categorized into the pro-inflammatory M1 and pro-regenerative M2 families. M1 macrophages, secreting inducible nitric oxide synthase (iNOS), induce muscle lysis, while M2 macrophages regulate satellite cell activity. Cytokines (TNFα, IFNγ) and interleukins (IL4, IL10) synergistically control the proliferation of M1 and M2 macrophages. In dystrophic mice, chronically activated intracellular signaling pathways result in a significant deregulation of these macrophage populations, leading to excessive tissue destruction and the recruitment of pro-inflammatory CD4/CD8+ T-cells [6].

Several investigative approaches have illuminated the multifaceted nature of DMD, unrevealing primary and secondary features that unfold in distinct phases due to the absence of dystrophin. Notably, adaptive immunity emerges as a secondary aspect, hinting at an environmental trigger for chronic muscle inflammation amid pre-existing innate immunity activation. The pathological manifestation of DMD is intricately linked to mutation types, DGC protein absence, and the extent of respiratory and cardiac involvement [7].

A wealth of data from diverse transcriptomic and genomic analyses underscores the pivotal role of genetic modifiers in predicting disease prognosis, closely aligning with clinical outcomes for DMD patients. Despite the potential clinical utility of categorizing these modifiers into distinct phenotypes, challenges persist in their classification. Desguerre, for instance, navigated the clinical heterogeneity of DMD by stratifying based on the severity of muscle and brain dysfunction [8]. Likewise, numerous studies spanning various domains have aimed to pinpoint potential biomarkers for assessment in DMD patients [9,10,11].

To expedite the translation of fundamental knowledge into clinical applications, the quest for novel biomarkers and disease targets gains significance. This endeavor holds the promise of enhancing our understanding and designing innovative therapeutic strategies, ultimately contributing to improved care for individuals grappling with these debilitating diseases.

## 2. Biomarkers Unveiling the Complexity of Duchenne Muscular Dystrophy

In the past decade, researchers have concentrated their efforts on strategies aimed at countering mutations in the dystrophin gene. Gene therapy, exon skipping machinery, and the transplantation of autologous genetically corrected stem cells have emerged as potential approaches, although with contradictory outcomes. Simultaneously, various subpopulations of stem cells with myogenic potential have been evaluated for muscle regeneration and modulation of inflammatory pathways in dystrophic backgrounds, aiming to avoid the formation of tumorigenic aggregates [12].

To assess the clinical benefits of these treatments, outcome measures and functional scales have been developed to evaluate pathology progression in treated patients. However, the high variability among individuals with DMD has posed challenges for regulatory approval [13]. In light of these challenges, the identification of biomarkers is crucial to decipher the most recognizable features of both normal and pathological conditions, allowing for more feasible disease management. Among different classes of biomarkers, predictive biomarkers are commonly considered to recognize patient-specific characteristics, such as genetic mutations, and are essential for designing and conducting clinical trials. Diagnostic biomarkers strengthen the presence of the pathology and are necessary for patient stratification. All efforts required to determine a single biomarker become incredibly complex when considering the synergy among multiple biomarkers. In DMD, leveraging different techniques developed in the last 20 years, the analysis of body fluids, such as serum and blood, has yielded a considerable number of reliable biomarkers.

The most commonly used biomarker to detect muscle damage is the serum activity levels of creatine kinase (CK), which are normally correlated with the abundance of other muscle-derived circulating proteins, such as myofibrillar proteins and lactate dehydrogenase [14]. In the blood of DMD patients, other upregulated molecules could be recognized as aldolase—which is necessary to destroy sugars and produce energy and is released into the bloodstream when the cells are disrupted—and myosin light chain 1/3, filamin C, and myomesin 3, which are proteins associated to sarcomeric contraction. Similarly, in the urine, it is possible to identify other over-expressed metabolites such as creatinine, biliverdin, and ferritin [15].

Given that dystrophic muscles are typically affected by alterations in fatty acid accumulation and thiol oxidation [16,17], other candidates among lipids and metabolites have been studied. For instance, glutamate, succinate, and glycerol are increased in mdx mice [18,19], along with cholesterol, whose upregulation dramatically affects membrane functions [20]. In DMD patients, different studies have reported alterations in creatine/creatinine ratios [21] and trimethyl ammonium/total creatine ratios [22], but further analyses are needed to correlate the levels of these molecules with disease symptoms and severity.

The increasing of oxidative stress is a hallmark of pathology; its upregulation depends on over-expression of inflammatory cells, dysfunctions of mitochondria, and sarcolemma ruptures, leading to upregulation of NAD(P)H and xanthine oxidase, nitric oxidase synthase (NOS1), and neuronal (n)NOS [23]. The accumulation of these molecules affects the function of proteins and lipids, leading to over-expression of malondialdehyde and isoprostanes [24] and protein carbonyls that derive from irreversible modification of the side chains of aminoacidic residues by lipid peroxidation end products [25,26]. Similarly, the neutrophils that are activated in the first wave of inflammation express abundantly the powerful antioxidant hypochlorous acid (HOCl), which causes damage to muscular tissues and can be measured through the detection of halogenated tyrosine [27]. Lastly, the oxidation of thiol (-SH) groups of cysteine residues is associated with the development of areas of necrosis and fatty tissue and the accumulation of lipofuscin [28]. In this way, the evaluation of proteins with thiol groups like those of cysteine 34 (Cys34) is a biomarker of oxidative stress and myonecrosis.

The miRNAs, associated with widespread functions such as apoptosis, skeletal muscle development and regeneration, and regulation of cellular proliferation, have been studied in the context of DMD. Since these processes are affected by the dystrophic background and miRNAs are released into circulation following adverse events [29], various studies have confirmed elevated levels of miR-1, miR-133, and miR-206 in dystrophic serum [30,31]. However, additional clinical studies are required to correlate miRNA sensitivity to disease progression [32]. Following the analysis of gene expression omnibus (GEO) datasets, Han and co-workers identified several genes that were differentially expressed between the skeletal muscle tissues of DMD patients and healthy volunteers. Notably, complement C3 (C3), osteopontin (SPP1), thymosin beta 10 (TMSB10), and transmembrane immune signaling adaptor (TYROBP) were strongly related to the upregulation of immune cell infiltration, suggesting their potential as therapeutic targets to modulate inflammatory features of dystrophic tissues [33]. In a similar context, Coenen-Stass et al. identified the upregulation of phosphoglycerate mutase 1 (PGAM1), troponin I, cardiac muscle (TNNI3), myoglobin (MB), troponin I, fast skeletal muscle (TNNI2), and L-lactate dehydrogenase B chain (LDHB) proteins in the serum of mdx mice compared to wild-type. These elevated protein levels were subsequently restored to wild-type levels following the restoration of dystrophin through the injection of Pip6a-PMO in mdx mice, suggesting their potential utility in assessing the efficacy of therapeutic interventions in dystrophinopathic patients [34].

## 3. Gut Microbiota as an Immune-Regulator and Disease-Modulator

The gut microbiota plays a pivotal role in shaping and regulating immune responses, and disruptions in gut microbial composition, known as dysbiosis, have been linked to various autoimmune and immune-mediated diseases [35]. The commensal population comprising the microbiota varies widely among individuals, influenced by immune responses in the gut and host genotypes/phenotypes [36]. The intricate interactions between the microbiota and intestinal immune cells are tightly regulated, and dysfunctions in this system can lead to chronic inflammatory states [37].

This regulation involves innate immune sensors, Toll-like receptors (TLRs), expressed by macrophages, lymphocytes, and dendritic cells (DCs) [38]. TLRs recognize microbial factors (microbe-associated molecular patterns, MAMPs) and initiate signaling cascades, activating pro-inflammatory molecules like NF-κB/MAP kinases [39,40]. The commensal microbiota influences the expression of TLRs in intestinal epithelial cells, and modifications in the microbiota can alter the immunogenic roles of TLRs, thereby influencing the pro-inflammatory phenotype of the intestinal mucosa [41]. The microbiota also plays a crucial role in developing and coordinating the function of lymphoid cells in the intestine. Intestinal T-cell responses are influenced by the types of microorganisms in the microbiota and, notably, by the relationship between the commensal microbiota and antigens or metabolites derived from food intake. Dietary metabolites can act as immune modulators, as seen in murine models of experimental autoimmune encephalomyelitis fed with a low protein/calorie diet, which ameliorated disease symptoms, increased Tregs, and reduced pro-inflammatory cytokine expression. This diet was subsequently used to treat patients with multiple sclerosis (MS) [42].

Microbiota modulation contributes to MS by influencing the expression of genes involved in DC maturation and in monocytes/T-cells-dependent pro-inflammatory signaling [43,44]. Additionally, the microbiota is implicated in the development of obesity and diabetes, linking mucosal alterations to systemic low-grade inflammation and altered muscular/adipogenic pathways [45]. The richness and quality of bacterial species in the gut microbiota directly affect the development of adiposity and inflammation in obese individuals [46,47,48]. Recent studies have also correlated the intestinal microbiota with lifetime cardiovascular risk [49]. These findings underscore the pressing need to identify strategies targeting the gut microbial ecosystem and conduct in-depth analyses of the functional relationship between food and microbiota composition to modulate various human diseases.

## 4. Probing the Role of Intestinal-Derived MAMPs in Skeletal Muscle Activation and Degeneration

Skeletal muscle expresses a TLR4 receptor that could be activated by circulating LPS from the gut microbiota [50]. TLR4 is up-regulated in mdx muscles, and its ablation allowed an amelioration of the dystrophic phenotype [51]. Although TLR4 expression in the intestinal mucosa is low, the amount of this protein is up-regulated in the IELs and in other cells of the intestinal barrier following inflammatory events [38]. Once it is recognized by a ligand, TLR4 dimerizes and activates the signaling cascades that lead to the activation of a pro-inflammatory response. In the MyD88-dependent pathway, MyD88 phosphorylates different kinases that, in turn, activate MAPKs, TRAF6-NFκB, and their inflammatory cascade. Upon activation of the MyD88-independent pathway, TLR4 triggers the expression of IFN-dependent genes.

Among the signaling molecules that regulate immune response according to metabolite expression, mTOR, the aryl hydrocarbon receptor (AHR), and the family of peroxisome-proliferator-activated receptors might have an impact on muscle physiology. mTOR regulates the differentiation capacity of T-helper cells and the development of DCs [52]. Interestingly, the progressive muscle weakness (atrophy) of mdx muscles is caused by activated ubiquitin proteasome and autophagy systems that promote protein breakdown and reduced Akt/mTOR activities, ultimately leading to impaired protein synthesis and therefore a continuous catabolic state. The inhibition of the protein complex mTOR/mTORC1/mTORC2 in skeletal muscles is responsible for metabolic and mitochondrial abnormalities responsible for the development of muscular pathologies [53]. AHRs have similar functions in T-cell maturation and their effector abilities.

The amount of *p-AMPK*—the master regulator of metabolic processes that regulate energy homeostasis, activation of fatty acid oxidation, and glucose uptake in muscle—is dramatically down-regulated by the gut microbiota. The consequence of AMPK activation is the up-regulation of carnitine-palmitoyl transferase-1 (CPT-1) [54]. On the other hand, the activation of *muscular AMPK* is also induced by SCFAs [52], whose fecal concentrations are sensibly increased in animals that underwent running exercise due to modifications in microbiota composition. Interestingly, SCFAs have been described to coordinate the expression of cytokines by Th cells and modulate inflammation [55]. Leptin is a cytokine that enhances the activity of the thymus and allows the development of Th1 cells. It is fundamental to maintain the organization of IELs, and their abundance is strictly related to the microbiota, as in GFM it is dramatically down-regulated [52]. Ghrelin (GHR) has opposite immunological functions compared to leptin, but together they are involved in inflammatory pathologies of the colon [56]. GHR stimulates food intake and adiposity [57] and, in skeletal muscles, attenuates skeletal muscle wasting both by up-regulating anabolic molecules (IGF-1, STAT5) and down-regulating proteolytic systems (activated-NFκB, FoxO1, MuRF1). GHR is expressed by T-cells/monocytes, reduces the expression of pro-inflammatory molecules [58], and activates mTOR/Akt signaling in atrophic muscle [59]. The lack of microbiota is responsible for the up-regulation of the *fasting-induced adipose factor* (*FIAF*) in the intestine, which inhibits the expression of genes whose function is strictly correlated to fatty acid muscular oxidation. FIAF is responsible for the over-expression of PGC1-α that primary regulates mitochondrial activity and the rate of expression of atrophy-related genes such as MuRF-1 and Atrogin-1 [60]. The gut microbiota regulates the availability of several amino acids and, more interestingly, metabolites that are implicated in energy formation and muscle fat deposition [61].

## 5. Gut Microbiota Shapes the Landscape of DMD Pathogenesis

While it is known that the intestinal wall of mdx mice is inflamed, exacerbating gastrointestinal dysfunctions, little is understood about the development of inflammation from the intestine and the role of different immune subpopulations. Interestingly, there is a growing recognition of nutrition-based approaches to modulating chronic inflammatory responses by influencing the gut microbiota [10]. In mdx mice, we demonstrated that nutraceutical supplementation limited the production of reactive oxygen species (ROS) and the recruitment of inflammatory cells in muscle tissues [11]. Additionally, supplementation with a branched-chain amino acid-enriched mixture partially inhibited the pathological phenotype [12]. Consistent with these findings, in dystrophic patients, oral administration of natural polyphenols reduced creatine phosphokinase (CPK) concentrations and the frequency of circulating inflammatory progenitors [13].

Based on these results, we proposed that dystrophic gastrointestinal inflammation is dependent on the interplay between the intestine and its microbiota, and this axis is responsible for disseminating inflammatory signals throughout the muscles. Furthermore, other regulators implicated in the modulation of inflammatory events in ulcerative colitis (UC) and Crohn’s disease (CD) patients, such as the IL-33/ST2 axis and the IP, were found to be commonly altered in DMD [62,63,64].

**Figure 1:**
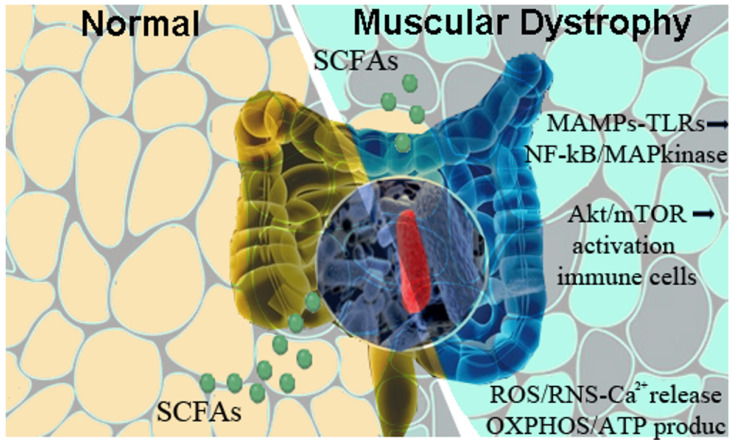
Reduction of richness in the intestinal microbial communities of mdx mice alters the expression of short-chain fatty acid (SCFA) biosynthetic enzymes and provokes modulations of innate immune sensors, the Toll-like receptors (TLRs). These sensors recognize microbe-associated molecular patterns (MAMPs), leading to activation of pro-inflammatory NF-κB/MAP kinases. Dysfunctions in mTOR/Akt pathways determine immune-mediated activation of inflammatory cells, while the excess of calcium ions causes the up-regulation of reactive oxygen species (ROS) and reactive nitrogen species (RNS) that affect mitochondria and ER functionality. The combination of these events contributes to the worsening of DMD pathological phenotype.

In line with these findings, we investigated the origin and principal participants of these phenomena (Figure 1).

It is well established that dystrophic patients experience gastrointestinal impairments, motility alterations, and smooth muscle fibrosis [65]. Similarly, the gastrointestinal tract of mdx mice exhibits nitric-oxide dysfunctions [66] and calcium overexpression [67], significantly impacting fecal output and propulsion [65]. Following these observations, we explored the richness of the intestinal microbial community and identified a significant reduction in microbial richness in dystrophic mice compared to healthy ones. Specifically, we observed an overexpression of the genera *Alistipes* and *Prevotella*, which positively correlated with the frequency of immune cell populations, such as splenic CD44+CD4+/CD8+ T cells and Tregs, as well as muscle effector/memory CD44+CD8+ T cells and central memory CD4+ T cells [68]. Other researchers demonstrated that over-expression of *Prevotella* is associated with alterations in metabolic and inflammatory conditions [69] as well as a pro-inflammatory state in elderly individuals [70]. Metabolic profile analysis in mdx mice revealed alterations in carbohydrate and amino acid metabolism pathways, along with a significant decrease in the expression of short-chain fatty acid (SCFA) biosynthetic enzymes. Intriguingly, we determined that partial or total depletion of the gut microbiota reduced the innate immune response and the expression of genes involved in early myogenesis, altering muscle metabolism, architecture, and force. Given that the colonization of mdx mice with the microbiota content of C57Bl mice resulted in a reduction in inflammatory development while improving muscular functions, we posited that the gut microbiota plays a pivotal role in the pathogenesis of DMD [68]. To validate our findings, Kalkan et al. demonstrated alterations in circulating SCFAs and ketone bodies (KBs) in mdx mice compared to C57Bl mice. Building upon these initial results, they found that treatment with sodium butyrate in the DMD murine model improved locomotor activity, autophagy mechanisms, and inflammation through the over-expression of endocannabinoid signals. Similarly, butyrate supplementation in myoblasts derived from DMD patients ameliorated inflammation and autophagy, potentially via the inhibition of micro-RNAs specific to cannabinoid-receptor 1 [71].

It is widely acknowledged that prednisone, the most commonly used glucocorticoid in DMD treatment, induces gut dysbiosis, exacerbating inflammation and impacting gut barrier functions in treated patients. Modulating the microbiota has been shown to counteract the side effects of this drug in animal models of inflammatory and autoimmune diseases [72,73], suggesting the potential of microbiota targeting as a viable therapeutic approach in DMD [74]. Interestingly, it was noted by Kalkan et al. that another glucocorticoid, deflazacort, positively impacts microbiota composition, SCFAs, and KBs content in mdx mice, making a contribution to ameliorating the pathological phenotype [71].

Considering that skeletal muscle regulates crucial functions such as glucose uptake, fatty acid oxidation, and protein metabolism [75], it was hypothesized that the gut microbiota may influence muscle-resident mitochondria, leading to alterations in innate immune cells, possibly through ROS, reactive nitrogen species (RNS), and various myokines such as IL-6 [76]. Consistent with these findings, increased autophagy could facilitate the elimination of dysfunctional mitochondria, mitigating muscle wasting and extending the lifespan of treated mice [77,78,79]. Additional studies have shown that reactive oxygen species (ROS) generated in a limited number of mitochondria can impact nearby mitochondria and other cellular organelles. Mitochondria may collaborate synergistically with other cellular sources of ROS, such as the endoplasmic reticulum (ER), creating a feedback loop [80]. During stress or oxidative enhancement, the upregulation of ROS driven by ER causes modulation of mitochondria, allowing the influx of calcium ions into the cytoplasm and increasing ROS production [81]. Since the endoplasmic reticulum (ER) plays a crucial role in mediating the folding of secretory proteins and the biosynthesis of sterols and fatty acids [82], the up-regulation of the GRP78 chaperone has been observed in mdx muscle. GRP78 serves as a marker for the accumulation of the unfolded protein response, impacting proper muscle contractility and promoting muscle cell death [83]. RNS can enhance the activity of ROS, facilitating the release of calcium from the ER and influencing various functions such as oxidative phosphorylation and ATP production from mitochondria [84].

## 6. Exploring Tdark Genes in Duchenne Muscular Dystrophy

In recent years, genetic strategies have been employed to address the vast array of dystrophin mutations, while cellular studies have focused on identifying subpopulations with the highest expansion/homing capacity. Unfortunately, only immunosuppressive drugs have managed to delay symptom onset, but their extensive use is hindered by serious adverse effects. Inflammatory features in DMD have heightened the immunogenicity of transgenes through gene transfer [85], yet clinical targets remain elusive. Advancements in genome sequencing have delved into poorly covered regions, revealing thousands of non-coding RNA sequences crucial for mRNA production, epigenetic programming, and subsequent protein translation.

**Figure 2:**
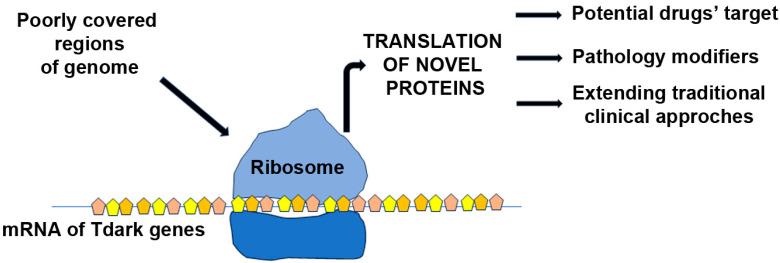
Advancements in techniques to sequence genomes deciphered enormous amount of non-coding RNA sequences crucial for mRNA production and subsequent protein translation. Among them, Tdark are those genes whose way of action and synergy with other molecules are still unknown or at least on-going. This way, Tdark genes represent newly therapeutic targets to characterize and augment their contribution to the management of DMD from a clinical point-of-view.

In 2017, the research group led by Oprea launched Pharos to enhance information on proteins recognized as potential drug targets [86]. Categorizing targets based on their “Target Development Level” (TDL), Tdark genes are those of which the mode of action or bound molecules are unknown [87]. Importantly, Tdarks represent unexplored opportunities for future drug development, as no chemical compounds exist that inhibit or limit their expression. In line, despite unsuccessful attempts with numerous genes as DMD clinical targets, Tdark genes represent unexplored therapeutic possibilities to understand and might reduce DMD disease progression (Figure 2).

According to the Knowledge Management Center (KMC) for the Illuminating the Druggable Genome (IDG) program—IDG-KMC—funded by the National Institutes of Health (NIH), there are 42 Tdark proteins associated with DMD.

Among them, *mxra7*, a matrix remodeling-associated (MXRA) gene [88], is co-expressed with genes coordinating cellular adhesion and extracellular matrix (ECM) remodeling. It is up-regulated in inflammation-induced neovascularization [89] and plays a role in tissue damage and regenerative processes [90,91,92]. MXRA7^ko^ mice down-regulate neutrophils, suppress cytotoxic lymphocytes and inflammatory cytokines, and modulate pathways such as those dependent on mitogen-activated protein kinase (MAPK) and protein kinase B (AKT)/NF-κB [93]. Intriguingly, *mxra7* is one of the most abundantly expressed genes in bone marrow-derived mesenchymal stem cells [93], simultaneously modulating genes expressed in the extra-ECM, such as matrix metalloproteinases, and being essential for fibroblast proliferation [94]. We recently demonstrated that the up-regulation of MXRA7 expression in dystrophic conditions could be dependent on a combination of different phenomena, such as the development of fibrosis and modulation of ECM components together with the dysfunctions of cellular composition, possibly related to aging.

Similarly, the *Leucine Rich Repeat Containing 17* (*lrrc17*) gene is expressed in the ECM, regulating the proliferation of cellular subpopulations in the bone marrow [95] and the differentiation of osteoclasts [96]. The *MAM Domain Containing 2* (*mamdc2*) gene, predicted to be an ECM protein, is expressed in myonuclei and sarcoplasm, regulating the glycosaminoglycan pathway [97]. *Glucoside Xylosyltransferase 2* (*gxylt2*) regulates the activity of the Notch pathway and determines the phosphorylation of MAPK, accelerating cell growth and migration of human cancer cells [98]. Intriguingly, other studies showed that *gxylt2* was correlated to negative prognosis and immune infiltration in bladder cancer [99], and its methylation was associated with epithelial cells in the inflamed colon of patients suffering from ulcerative colitis [100]. *Transcription elongation factor A (SII)-like 9* (*tceal9*) and *8* (*tceal8*) modulate transcription and protein-protein interactions [101]. *Nipsnap Homolog 3A* (*nipsnap3a*) is involved in vesicular transport [102] and—together with other proteins in the family—participates in mitophagy and mitochondrial metabolism while modulating immune responses [103]. *Ribosomal Protein L22 Like 1* (*rpl22l1*) is thought to be a component of the ribosome where it mediates the functions of Nodal Growth Differentiation Factor (Nodal)/TGF-β signaling as well as *small mother against decapentaplegic* (*smad*) expression [104]. Indeed, *rpl22l1* takes part in the regulation of CCL2 and—more in general—of the immune responses that resolve tissutal inflammation [105]. Given that these functions and, in particular, inflammation are commonly altered in mdx mice and DMD patients, exacerbating pathogenesis, further studies are warranted to elucidate the role of Tdarks in this disease.

## 7. Discussion

The recent strides in genetic strategies for addressing dystrophin mutations in DMD have opened new avenues, yet the clinical landscape is marked by challenges. Cellular studies, with a focus on identifying subpopulations with high expansion/homing capacity, complement these genetic approaches [106]. Despite these efforts, corticosteroids remain the only intervention capable of delaying symptom onset, albeit with substantial limitations due to serious adverse effects.

Multiple lines of investigation revealed that DMD is characterized by primary and secondary features caused by dystrophin absence that occurs in sequential phases. Notably, adaptive immunity is one of the secondary features of DMD, which proposes that an environmental factor triggers chronic muscle inflammation in the context of pre-existing innate immunity activation. Although a role for the gut microbiota has been clearly established in muscle homeostasis [75], it is not known if dysbiosis influences DMD and how it modulates the pathophysiology. Our preliminary data suggest exhaustively that residing gut microbial communities could be implicated in DMD progression, influencing clinical and phenotypic variability in dystrophic patients by modulating metabolic and immune responses. All in all, the balance of microbiota composition is crucial to maintaining correct muscle function, and the GF murine models retain both beneficial and deleterious effects [107]. The exploration of the gut-muscle axis has been predominantly centered around animal models in current literature; however, the significance of gut-microbial status is not limited to the realm of animal studies as it extends to athletic humans and individuals facing muscle-wasting conditions like sarcopenia and cachexia [108,109]. In this context, the gut microbiome emerges as a potential biomarker of human health. Existing evidence underscores the possibility of implementing a combined pro- and prebiotic regimen. This approach aims to shape the gut microbiota with an optimal microbial profile while providing suitable substrates, thereby addressing muscle wasting through positive modulation of the gut microbiota. The integration of both pro- and prebiotics acknowledges the symbiotic relationship between these microbial agents and the host. While the full extent of the impact on muscular dystrophies remains uncertain, there is promise in the idea that microbe-based therapeutics, inspired by successful animal studies, could potentially translate into human applications. The prospect of mitigating muscle wasting and extending life expectancy becomes particularly encouraging when considering the potential synergy with established interventional strategies such as prednisone treatment and exercise. In essence, the gut-muscle axis represents a dynamic and interconnected system that influences not only animal models but also human health, especially in conditions characterized by muscle wasting [74,110].

The evolving understanding of the gut microbiome as a biomarker opens up avenues for innovative therapeutic approaches. While challenges and uncertainties persist, the potential benefits for individuals facing muscle-related disorders are substantial, offering hope for improved outcomes and enhanced quality of life. Further research and clinical investigations will be crucial to disclose the full therapeutic potential and practical implications of targeting the gut microbiome in the context of muscle health. In the quest for effective therapeutic targets, the inflammatory features of DMD have brought attention to the immunogenicity of transgenes through gene transfer [111]. This revelation underscores the need for alternative clinical targets. Genome sequencing advancements, particularly in poorly covered regions, have unveiled thousands of non-coding RNA sequences critical for mRNA production and protein translation.

The human genome was sequenced twenty years ago, but its exact gene composition remains a subject of debate, and the number of protein-coding genes is much lower than initially expected. The majority of the untranslated genome is fundamental—among the others—to regulate genes’ expression, control protein folding, and modulate the transcriptome in response to all the environmental pressures. Intriguingly, it became clear that this understudied portion of the genome could be an incredible source for drug discovery analysis to identify feasible targets or novel genes associated with physiological processes such as autophagy or correlated to cancer development [87]. One noteworthy classification arising from this effort is the identification of Tdark genes, whose interactions with other molecules as well as their functions in pathophysiological mechanisms are still unknown or at least on-going to be deciphered. Despite previous unsuccessful attempts with numerous genes as DMD clinical targets, Tdark genes represent uncharted therapeutic possibilities, as the dysfunctions of some of them lead to pathological signs commonly altered in dystrophic patients. Investigating MXRA7 as a potential blood/serum biomarker for fibrosis in DMD patients, along with identifying the specific cellular subpopulation(s) responsible for its expression, holds significant promise to enhance our comprehension of fibrotic processes and, more importantly, to develop clinical strategies to counteract fibrosis.

These findings pave the way for a more nuanced understanding of DMD and the potential development of targeted therapeutic interventions that extend beyond traditional approaches.
